# Supplementary material for: Information-Seeking Decision Strategies Mitigate Risk in Dynamic, Uncertain Environments
Source: ArXiv. 2025 Mar 24:arXiv:2503.19107v1. Preprint. [Version 1] (PMC11975046)
Supplement: Supplement 1 [file NIHPP2503.19107v1-supplement-1.pdf]

A Phase Transition Approximation

Here we detail the calculation for approximating the explore-exploit phase transition's location as shown in Fig. 2. For our approximation, we compare the average change in belief magnitude from feedback versus sampling and assume that the agent takes whichever action leads to the largest magnitude change when either state is equally likely (i.e., for LLR belief $y = 0$). This approximation can be thought of as choosing the most "impactful" action on the very first action of the sequence, and if it is most impactful for the agent

to commit to a decision, they will likely continue making commitments for the remainder of their action sequence. Throughout these calculations, we use the notation Y_n^i to represent the random variable for the agent's LLR belief after observing n environmental samples in the i-th environment, and y_n^i to represent the realized LLR value, similar to the notation used throughout Section 2.

To calculate the average LLR magnitude change associated with feedback, we start by using the Law of Total Probability to condition the magnitude change over the decision d^i and feedback r^i:

$$\mathbb{E}\left[|Y_0^{i+1} - Y_n^i||Y_n^i = y_n^i = 0\right] = \sum_{d^i \in \{s_+, s_-\}, r^i \in \{\circ, \times\}} \mathbb{E}\left[|Y_0^{i+1}||y_n^i = 0, d^i, r^i\right] \Pr\left(d^i, r^i | y_n^i = 0\right). \quad (19)$$

Next, we note that, given the conditioning on y_n^i, d^i, and r^i, the LLR belief Y_0^{i+1} is fixed and given by Eq. (5). Inspecting Eq. (5), we can see that the LLR belief Y_0^{i+1} is the same regardless of r^i and d^i, allowing us to pull this expectation out of the sum in Eq. (19). At this point, the remaining sum of probabilities equals unity, because the sum contains all possible realizations of the joint event (r^i, d^i). Performing these simplifications yields the expected change in LLR magnitude:

$$\mathbb{E}\left[|Y_0^{i+1} - Y_n^i||Y_n^i = y_n^i = 0\right] = \left|\ln \frac{(1-\epsilon)q + \epsilon(1-q)}{\epsilon q + (1-\epsilon)(1-q)}\right|. \quad (20)$$

To calculate the expected change in LLR magnitude associated with environmental sampling, we first define this magnitude change using Eq. (1) as:

$$|Y_{n+1}^i - Y_n^i| = \left|\ln \frac{f_+(\xi_{n+1})}{f_-(\xi_{n+1})}\right|. \quad (21)$$

Using the fact that ξ_{n+1} follows the Bernoulli distribution given by Eq. (10) allows us to simplify this magnitude change:

$$|Y_{n+1}^i - Y_n^i| = \begin{cases} \left|\ln \dfrac{h}{1-h}\right|, & \xi_{n+1} = +1 \\ \left|\ln \dfrac{1-h}{h}\right|, & \xi_{n+1} = -1 \end{cases},$$

which, due to logarithm rules, is constant for all possible realizations of ξ_{n+1} and is independent of the agent's current LLR belief value:

$$|Y_{n+1}^i - Y_n^i| = \left|\ln \frac{h}{1-h}\right|. \quad (22)$$

From Eq. (20) and Eq. (22), we obtain our approximation: the phase transition occurs when the expected LLR magnitude change from feedback exceeds the expected LLR magnitude change from sampling, which implies the phase transition's boundary is where these two quantities are equal:

$$\left|\ln \frac{(1-\epsilon)q + \epsilon(1-q)}{\epsilon q + (1-\epsilon)(1-q)}\right| = \left|\ln \frac{h}{1-h}\right|. \quad (23)$$

For the limits of environmental parameters ($\epsilon \in [0, 0.5]$, $q \in [0.5, 1]$) and evidence-generating parameters ($h \in [0.5, 1]$) we considered, both logarithms in Eq. (23) output non-negative values, allowing us to make a further simplification by dispensing of the absolute values, producing Eq. (17):

$$(1-h)\left[(1-\epsilon)q + \epsilon(1-q)\right] = h\left[\epsilon q + (1-\epsilon)(1-q)\right].$$

B Supplemental Figures

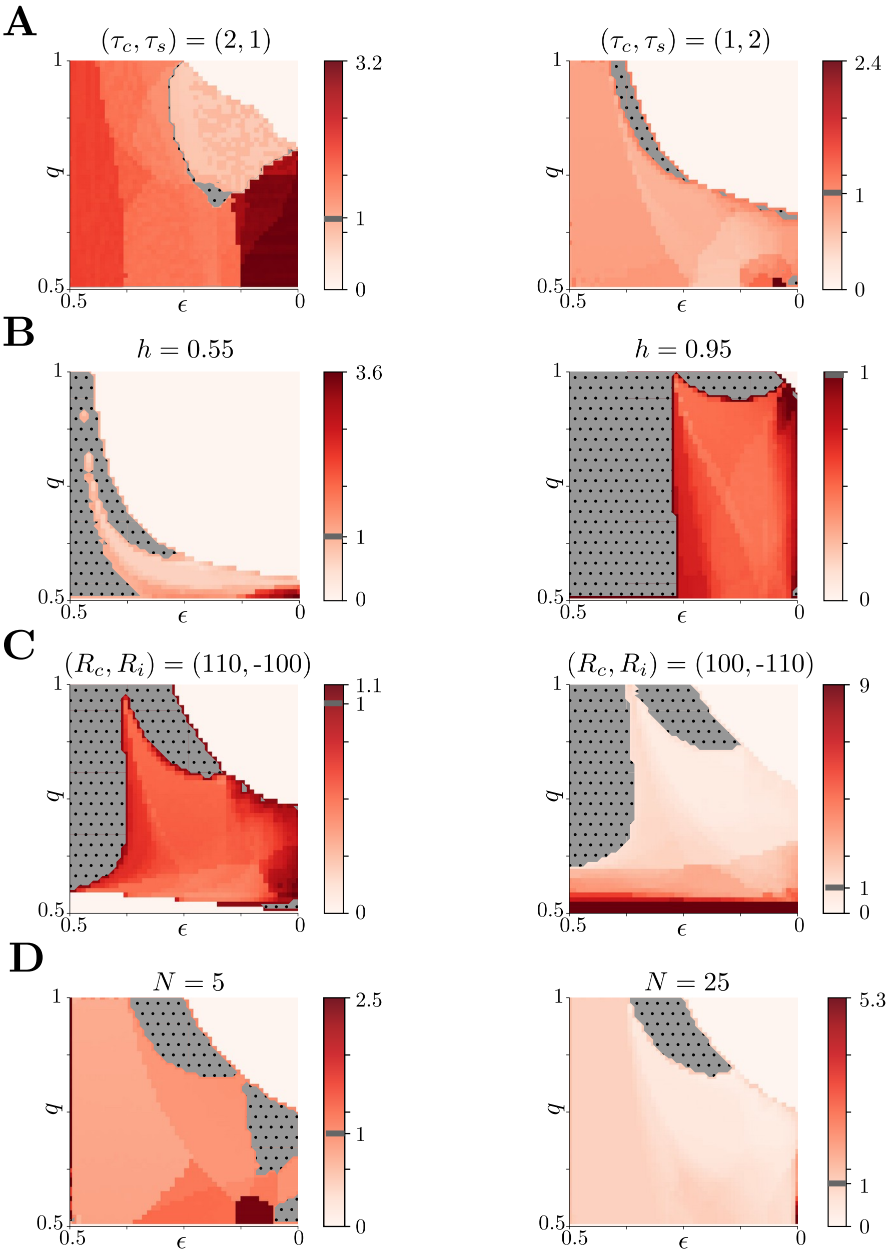

Figure Sup-1: **Rewardmax Phase Transition Behavior.** **A:** Ratio of average sample burst length to average commit burst length for rewardmax behavior as in Fig. 2**C**, but with action time step costs $(\tau_c, \tau_s) = (2, 1)$ (left) and $(\tau_c, \tau_s) = (1, 2)$ (right). **B:** Same as **A**, but for environmental evidence Bernoulli parameter $h = 0.55$ (left) and $h = 0.95$ (right). **C:** Same as **A**, but for reward structure $(R_c, R_i) = (110, -100)$ (left) and $(R_c, R_i) = (100, -110)$ (right). **D:** Same as **A**, but for action budget $N = 5$ (left) and $N = 25$ (right). Unless specifically altered, all other task parameters are the same as in Fig. 2.

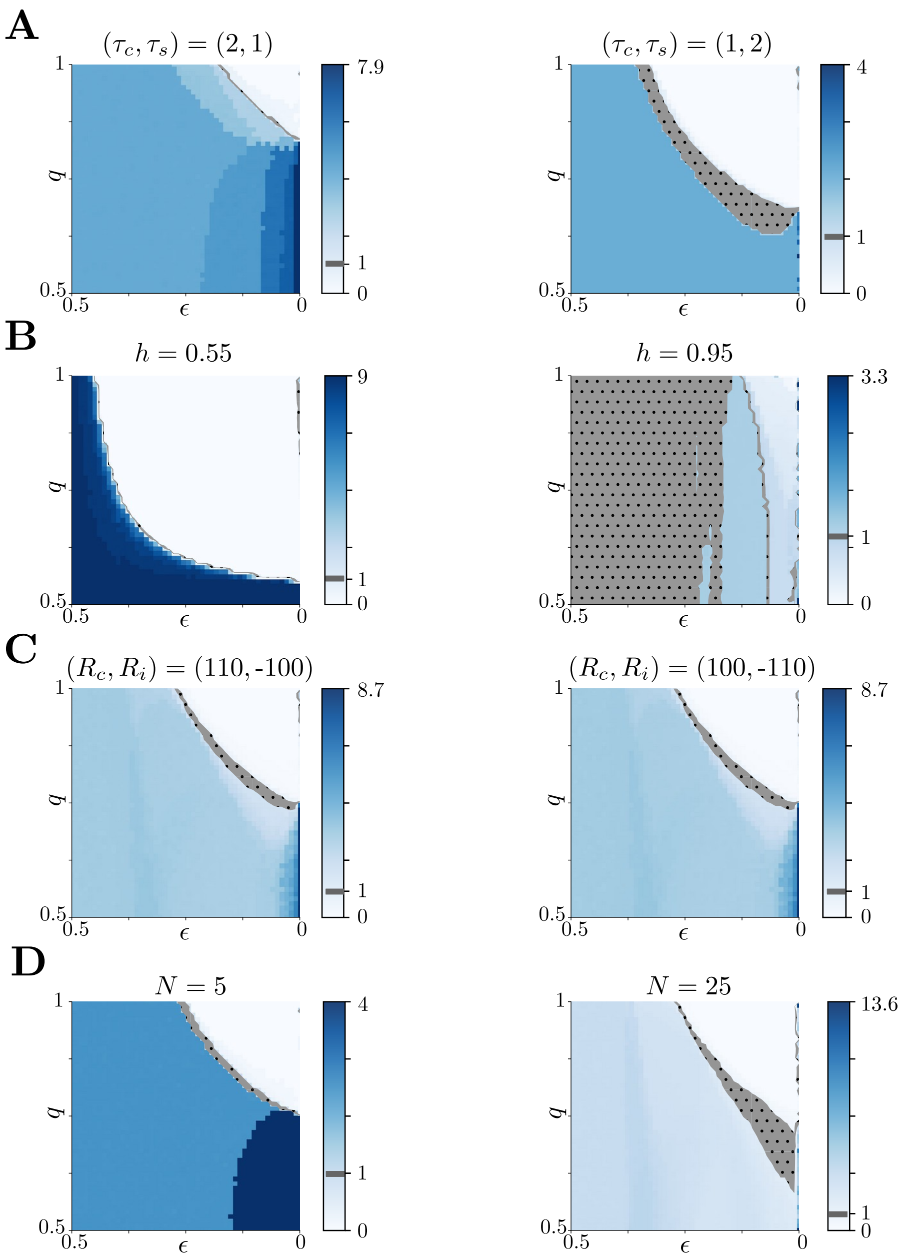

Figure Sup-2: **Infomax Phase Transition Behavior.** **A:** Ratio of average sample burst length to average commit burst length for infomax behavior as in Fig. 2**D**, but with action time step costs $(\tau_c, \tau_s) = (2, 1)$ (left) and $(\tau_c, \tau_s) = (1, 2)$ (right). **B:** Same as **A**, but for environmental evidence Bernoulli parameter $h = 0.55$ (left) and $h = 0.95$ (right). **C:** Same as **A**, but for reward structure $(R_c, R_i) = (110, -100)$ (left) and $(R_c, R_i) = (100, -110)$ (right). **D:** Same as **A**, but for action budget $N = 5$ (left) and $N = 25$ (right). Unless specifically altered, all other task parameters are the same as in Fig. 2.
